# The effect of interaction between hepatitis C virus and cigarette smoking on the risk of hepatocellular carcinoma

**DOI:** 10.1038/sj.bjc.6602981

**Published:** 2006-02-07

**Authors:** Y Fujita, A Shibata, I Ogimoto, Y Kurozawa, T Nose, T Yoshimura, H Suzuki, N Iwai, R Sakata, S Ichikawa, A Tamakoshi

**Affiliations:** 1 Department of Public Health, Kurume University School of Medicine, Kurume 830-0011, Japan; 2 Department of Social Medicine, Division of Health Administration and Promotion, Faculty of Medicine, Tottori University, Yonago 683-8503, Japan; 3 Fukuoka Institute of Health and Environmental Sciences, Dazaihu 818-0135, Japan; 4 Department of Public Health, Faculty of Medicine, Niigata University School of Medicine, Niigata 951-8510, Japan; 5 Chugoku Occupational Health Association, Tottori 680-0942, Japan; 6 Department of Preventive Medicine/Biostatistics and Medical decision Making, Nagoya University Graduate School of Medicine, Nagoya 466-8550, Japan

**Keywords:** HCC, HCV, JACC study, epidemiology, Japanese

## Abstract

We evaluated the interaction between hepatitis C virus (HCV) and cigarette smoking on death from hepatocellular cancer in The Japan Collaborative Cohort Study. The odds ratio of death from HCC for smoking was 9.60 (1.50–61.35) and 1.71(0.58–5.08) among anti-HCV positive and negative individuals, respectively.

Many studies have reported that chronic hepatitis C virus (HCV) infection is a risk factor for hepatocellular carcinoma (HCC) (Mori *et al*, 2000; El-Serag, 2002; Sun *et al*, 2003; Ayoola and Gadour, 2004), HCV appearing to be more hepatocarcinogenic than hepatitis B virus (HBV) (Pang *et al*, 2005). While alcohol is a well established risk factor for HCC, there is evidence that cigarette smoking is also a risk factor (Mukaiya *et al*, 1998; Mizoue *et al*, 2000; Chen *et al*, 2003), though some studies reported no or an insignificant association (La Vecchia *et al*, 1988; Evans *et al*, 2002). Recently, an association between diabetes mellitus and HCC (or HCV) has been reported (Caronia *et al*, 1999; Toyoda *et al*, 2001). We evaluated the interaction between HCV infection and cigarette smoking by means of a nested case–control study from a large cohort.

## MATERIALS AND METHODS

The Japan Collaborative Cohort Study (JACC Study) for Evaluation of Cancer Risk sponsored by the Ministry of Education, Science, Sports and Culture of Japan (Monbusho) is a large prospective cohort study, which was mounted from 1988 to 1990 in 45 areas in Japan. The number of subjects is 110 792 (46 465 males and 64 327 females) who were 40–79 years of age at the time of the baseline survey. Individual informed consent to participate in the study was obtained in 36 out of 45 areas (Tamakoshi *et al*, 2005). The subjects were asked to complete a self-administered questionnaire about past medical history, various life style factors and marital status as baseline information. The detailed design of the JACC study has been described previously (Ohno and Tamakoshi, 2001; Watanabe *et al*, 2005). During the approximately 10 years of follow-up through December 31, 1999, there were 550 deaths from liver cancer that were coded as C22 in the International Classification of Diseases and Related Health Problems, 10th Revision. Those survey participants who underwent health-screening checks sponsored by municipalities were asked to donate blood samples during the same period as the questionnaire survey. Eventually, 39 242 subjects provided blood samples (Tamakoshi *et al*, 2005). Baseline serum samples had been collected 120 of the 550 subjects who died of liver cancer. As the control group, sera of 11 543 subjects from the same geographical areas as the 120 deaths also were screened for anti-HCV. Deaths (nine) coded as C22.9 (liver cancer not otherwise specified) were excluded from this analysis, while deaths (five) coded as C22.1 (intrahepatic cholangiocarcinoma and cholangiohepatoma; included among non-HCC deaths), leaving 106 deaths from HCC that were regarded as the end point of this analysis. The total subjects were 11 654 but as the sera of 34 cases and controls could not be screened because of insufficient serum volume, the sera of 11 620 subjects were screened for antibody to HCV (anti-HCV) and then divided into anti-HCV positiveand anti-HCV negative groups. In each group, the cases were deaths due to HCC. The controls (living) were individually matched with cases for age (±3 years), gender and area, the cases or the controls that could not be matched being eliminated from this analysis; in the end, there were 3431 subjects.

We used SAS version 8.2 software (SAS institute, Cary, NC, USA) for the statistical analysis. Baseline information on smoking habits was divided into three groups. Matched multivariate-adjusted odds ratios (OR) and 95% confidence interval (CI) for risk factors for death due to HCC were estimated after adjusting for potential confounding factors (alcohol-drinking habit, past history of liver disease and past history of diabetes mellitus) using a conditional logistic model. With respect to interaction between HCV and smoking habits on HCC risk, we evaluated this by examining whether the odds ratio of death from HCC for each factor differed between anti-HCV positive group and anti-HCV negative group.

## RESULTS

Table 1 provides details of the HCC cases and matched controls by cigarette smoking habits. Subjects who lacked data on the adjusted factor were eliminated from the multivariate analysis (Table 2). In the anti-HCV positive group, the OR of death due to HCC was 7.84 (95% CI: 1.09–56.05) for ex-smokers and 9.60 (95% CI: 1.50–61.36) for current smokers. The OR for ‘<35’ and ‘35 or more’ of smoking period were 11.02 (95% CI: 1.67–72.64) and 6.99 (95% CI: 1.03–47.51), respectively. The OR for smoking on average ‘10 to 19 and ‘20 or more’ cigarettes per day were 12.47 (95% CI: 1.82–85.56) and 9.10 (95% CI: 1.10–75.05), respectively. We found no significant association between HCC and smoking period or average number in anti-HCV negative group.

## DISCUSSION

There are a few reports evaluating the joint effect of HCV infection and smoking habits on HCC. Sun *et al* (2003) suggest that the adjusted relative risk of HCC development was 3.9 for smokers who were positive for anti-HCV in comparison with that for nonsmokers who were negative for anti-HCV. Yu *et al* (1991) reported that there were significantly synergistic effects of anti-HCV with cigarette smoking. We performed multivariate analysis of an interaction between smoking habits and HCV on death from HCC controlling the potential confounding factors in the JACC study. Our results showed that cigarette smoking was associated with significantly elevated risk of developing HCC only among anti-HCV positive individuals. Much evidence indicates that the initiation or progression of HCC is a multistage process in which many factors are involved (Durr and Caselmann, 2000). The effect of cigarette smoking on persons with anti-HCV may involve promoting the progression from hepatitis to cirrhosis or from cirrhosis to HCC. In conclusion, we had observed an interaction between HCV infection and cigarette smoking on risk of death from HCC.

## Figures and Tables

**Table 1:** Distribution of the HCC cases and matched controls by smoking habits, smoking period and smoking average number

	**Anti-HCV positive group**		**Anti-HCV negative group**	
	**Case (%)**	**Control (%)**	**Control (%)**	**Case (%)**
*Smoking habits*				
Never smoker	13 (22.4)	241 (56.0)	16 (44.4)	1712 (58.9)
Ex smoker	8 (13.8)	54 (12.6)	5 (13.9)	378 (13.0)
Current smoker	30 (51.7)	116 (27.0)	15 (41.7)	688 (23.7)
Unknown	7 (12.1)	19 (4.4)	0 (0.0)	129 (4.4)
				
Total	58 (100.0)	430 (100.0)	36 (100.0)	2907 (100.0)
				
*Smoking period (year)*				
Never smoker	13 (27.1)	241 (60.9)	16 (44.4)	1712 (63.0)
<35	13 (27.1)	54 (13.6)	6 (16.7)	467 (17.2)
35 or more	22 (45.8)	101 (25.5)	14 (38.9)	538 (19.8)
				
Total	48 (100.0)	396 (100.0)	36 (100.0)	2717 (100.0)
				
*Smoking average number (cig/day)*				
Never smoker	13 (26.5)	241 (59.2)	16 (45.7)	1712 (62.2)
<10	0 (0.0)	13 (3.2)	1 (2.9)	85 (3.1)
10 to 19	13 (26.5)	49 (12.0)	6 (17.1)	336 (12.2)
20 or more	23 (47.0)	104 (25.6)	12 (34.3)	618 (22.5)
				
Total	49 (100.0)	407 (100.0)	35 (100.0)	2751 (100.0)

**Table 2:** Odds ratio (OR) of death from HCC by characteristics of smoking habits, smoking period and smoking average number

	**Anti-HCV positive group**				**Anti-HCV negative group**			
	**Case**	**Control**	**OR^a^**	**95% CI**	**Case**	**Control**	**OR^a^**	**95% CI**
*Smoking habits*								
Never smoker	13	220	1.00		13	1604	1.00	
Exsmoker	7	44	7.84	1.09–56.05	2	339	0.28	0.05–1.71
Current smoker	23	104	9.60	1.50–61.36	14	623	1.71	0.58–5.08
								
*Smoking period (year)*								
Never smoker	13	220	1.00		13	1604	1.00	
<35	11	46	11.02	1.67–72.64	3	425	0.49	0.10–2.33
35 or more	16	89	6.99	1.03–47.51	13	488	2.41	0.63–9.21
								
*Smoking average number (cig/day)*								
Never smoker	13	220	1.00		13	1604	1.00	
<10	0	11	—	—	1	76	1.00	0.11–8.84
10 to 19	12	45	12.47	1.82–85.56	4	303	0.84	0.21–3.38
20 or more	16	89	9.10	1.10–75.05	10	556	1.27	0.38–4.20

a Adjusted for area, age, gender, past history of diabetes mellitus, past history of liver diseases and alcohol drinking habits.

## Appendix A

### JAPAN COLLABORATIVE COHORT STUDY GROUP

The present investigators involved, with the coauthorship of this paper, in the JACC Study and their affiliations are as follows: Dr Akiko Tamakoshi (present chairman of the study group), Nagoya University Graduate School of Medicine; Dr Mitsuru Mori, Sapporo Medical University School of Medicine; Dr Yutaka Motohashi, Akita University School of Medicine; Dr Ichiro Tsuji, Tohoku University Graduate School of Medicine; Dr Yosikazu Nakamura, Jichi Medical School; Dr Hiroyasu Iso, Institute of Community Medicine, University of Tsukuba; Dr Haruo Mikami, Chiba Cancer Center; Dr Yutaka Inaba, Juntendo University School of Medicine; Dr Yoshiharu Hoshiyama, University of Human Arts and Sciences; Dr Hiroshi Suzuki, Niigata University School of Medicine; Dr Hiroyuki Shimizu, Gifu University School of Medicine; Dr Hideaki Toyoshima, Nagoya University Graduate School of Medicine; Dr Kenji Wakai, Aichi Cancer Center Research Institute; Dr Shinkan Tokudome, Nagoya City University Graduate School of Medical Sciences; Dr Yoshinori Ito, Fujita Health University School of Health Sciences; Dr Shuji Hashimoto, Fujita Health University School of Medicine; Dr Shogo Kikuchi, Aichi Medical University School of Medicine; Dr Akio Koizumi, Graduate School of Medicine and Faculty of Medicine, Kyoto University; Dr Takashi Kawamura, Kyoto University Center for Student Health; Dr Yoshiyuki Watanabe, Kyoto Prefectural University of Medicine Graduate School of Medical Science; Dr Tsuneharu Miki, Graduate School of Medical Science, Kyoto Prefectural University of Medicine; Dr Chigusa Date, Faculty of Human Environmental Sciences, Mukogawa Women's University; Dr Kiyomi Sakata, Wakayama Medical University; Dr Takayuki Nose, Tottori University Faculty of Medicine; Dr Norihiko Hayakawa, Research Institute for Radiation Biology and Medicine, Hiroshima University; Dr Takesumi Yoshimura, Fukuoka Institute of Health and Environmental Sciences; Dr Akira Shibata, Kurume University School of Medicine; Dr Naoyuki Okamoto, Kanagawa Cancer Center; Dr Hideo Shio, Moriyama Municipal Hospital; Dr Yoshiyuki Ohno, Asahi Rosai Hospital; Dr Tomoyuki Kitagawa, Cancer Institute of the Japanese Foundation for Cancer Research; Dr Toshio Kuroki, Gifu University; and Dr Kazuo Tajima, Aichi Cancer Center Research Institute.
